# Person-Centred Palliative Home Care From a Patient and Family Carer Perspective – A Qualitative Interpretive Meta Synthesis

**DOI:** 10.1177/23333936261435388

**Published:** 2026-04-06

**Authors:** Lotta Pham, Malin Bengtsson, Stina Nyblom, Joakim Öhlén, Jeanette Källstrand

**Affiliations:** 1 Palliative Centre, Sahlgrenska University Hospital, Region Västra Götaland, Gothenburg, Sweden; 2 Institute of Health and Care Sciences, Sahlgrenska Academy, University of Gothenburg, Gothenburg, Sweden; 3 Centre for Person-Centred Care, University of Gothenburg, Gothenburg, Sweden; 4 Institute of Medicine, Sahlgrenska Academy, University of Gothenburg, Gothenburg, Sweden; 5 School of Health and Welfare, Halmstad University, Halmstad, Sweden

**Keywords:** palliative care, home care services, home nursing, patients, caregivers

## Abstract

With an ageing population and increasing chronic illness, the need for palliative care is growing. Most people prefer to be cared for at home when possible, making it essential to understand the experiences of patients and families receiving care at home to ensure it aligns with their needs. This study aims, therefore, to synthesise the literature regarding patients’ and family carers’ experiences of palliative care at home. This systematic review employed the 6S model as an analytical lens, with an abductive approach to the interpretive qualitative meta-synthesis. A total of 6,080 unique citations were yielded by searches conducted in PubMed, Embase, CINAHL, PsycInfo and Scopus. The synthesis included a total of 19 studies, of which 9 explored patient experiences, and 10 addressed both patients’ and family carers’ perspectives on palliative care at home. Three interconnected enablers were identified: accessible palliative care, trusting relationships, and maintaining everyday life at home. These demonstrate how palliative care can help patients and family carers feel safe and supported at home by ensuring accessible care through reliable professional support, promoting trusting relationships grounded in collaboration and open dialogue, and helping patients and family carers maintain everyday life through empowerment and person-centred care.

## Introduction

Healthcare systems worldwide face an increased need for palliative care as the global population is ageing and a rising number of individuals struggle with chronic, progressive illnesses (Connor, 2020). Palliative care is defined by the World Health Organization (WHO, 2020) as an approach that seeks to relieve suffering and enhance quality of life for both patients and their families who are facing challenges associated with life-threatening illness. Palliative care addresses a broad spectrum of needs by providing holistic care with a person-centred approach (WHO, 2020). Therefore, patients approaching end of life may benefit from palliative care independently of age, diagnosis, or prognosis (Etkind et al., 2017). Palliative care has been suggested to be beneficial from diagnosis to death, sometimes concurrently with disease modifying treatment (Murray et al., 2017).

In a number of countries, the desired model for place of care is increasingly shifting from institutional to home-based, with home often the preferred place of care and death among both patients and family carers (Fereidouni et al., 2021; Gomes et al., 2015; O’Sullivan et al., 2024). A primary motivation for this is that feeling at home has been described as central to palliative care, as it enhances the sense of comfort and security for individuals receiving care in their own environment (Dekkers, 2009). This feeling is related to wellness despite illness and disease, and encompass being safe, being connected and being centred (Öhlén et al., 2014). At-homeness, particularly among older people, is closely linked to being oneself and maintaining a sense of connection with close ones and familiar surroundings (Saarnio et al., 2016).

Systematic reviews suggests that palliative care provided in the home is associated with positive patient outcomes, such as improved quality of life, greater satisfaction with care, and an increased likelihood of dying at home, without compromising symptom management (Pask et al., 2025; Pinto et al., 2024). Appropriate and timely support from the healthcare system has been described as crucial when palliative care is provided in the home, as a “sense of security” and trust in professionals are important to promote wellbeing for both the family carers and the dying person (e.g. Morris et al., 2015; K. Stajduhar et al., 2010). In a meta-ethnography, Sarmento et al. (2017) synthesised patient and family carer experiences of palliative home care, identifying the presence and competence of health professionals as overarching components. Both of these components presuppose access to palliative home care services in order to enhance patients’ and family caregivers’ sense of security and preparedness for death at home (Sarmento et al., 2017). However, there are inequities of access to palliative care, as it is estimated that only around 14% of people receive palliative care worldwide, and access is largely confined to high-income countries (Peeler et al., 2025).

Although home is often described as the preferred place of care and death, current research gives limited insight into how palliative care at home is experienced by patients in relation to preserving self-image and sustaining a sense of at-homeness and security. Insight into everyday factors and care practices that enable palliative home care from the perspectives of patients and family carers is also limited. A deeper understanding of their experiences of receiving palliative care at home is therefore essential in order to better tailor palliative home care to their needs and preferences. The aim of this study is to synthesise the literature regarding patients’ and family carers’ experiences of palliative care at home. The research question was: what factors and practices enable palliative home care, from the perspective of patients and family carers?

## Theoretical Framework

The 6S model of person-centred palliative care, which conceptualises key dimensions and care needs at the end of life, provides a theoretical framework for exploring experiences of receiving palliative care at home. The 6S-model consists of six concepts representing different dimensions and care needs: Self-Image, Symptom Relief, Self-Determination, Social Relationships, Synthesis and Strategies (Henoch & Österlind, 2019). *Self-image*, which is frequently used interchangeably with the term *identity*, is the central concept of the model. It encompasses the patient as a person with biography and experiences (Österlind & Henoch, 2021). An important issue for health care is to support patients in maintaining everyday life, as this facilitates preservation of self-image (Henoch & Österlind, 2019). *Symptom relief* refers to bodily distress and care, with optimal symptom relief described as central to preserving self-image. *Self-determination* concerns autonomy and emphasises the importance of maintaining control and being involved in care decisions. This also includes handing over care decisions to a proxy decision-maker, such as a trusted close one or healthcare professional (Österlind & Henoch, 2021). *Social relationships* emphasise the value of connections with significant others, including family carers, but also the importance of building relations with healthcare professionals (Henoch & Österlind, 2019). The two concepts *Synthesis* and *Strategies* both refer to the existential needs that may actualise near death and are closely intertwined. While *Synthesis* refers to reflection over situations and experiences and looks back on life, *Strategies* concerns the life remaining (Österlind & Henoch, 2021).

The 6S model has been developed to support patients to live as well as possible at end of life (Österlind & Henoch, 2021) and is grounded in Weisman’s (1988) six concepts of appropriate death: care, control, composure, communication, continuity and closure. Adapted to nursing practice in Sweden, the model emphasises that care is co-created through a partnership between the dying person (who contributes experiences, knowledge, beliefs and preferences) and healthcare professionals (who contribute scientific knowledge and clinical expertise). In this way, the person becomes an active co-creator of care that aims to be appropriate and meaningful at the end of life (Österlind & Henoch, 2021). The model aims to support planning, documentation, and evaluation of palliative care (Henoch & Österlind, 2019).

## Methods

This systematic review employed an interpretive approach to a qualitative meta-synthesis of previously published peer-reviewed qualitative study results (Thorne et al., 2004). The reporting of the review was structured according to the ENTREQ statement (Tong et al., 2012). Ethical approval was not required for this literature review, which was conducted in accordance with principles of scientific integrity.

### Literature Search and Selection

In April 2025, literature searches were conducted by LP and MB in collaboration with a university expert librarian. The set of databases searched included PubMed, Embase, CINAHL, PsycInfo, and Scopus. All records were identified through electronic searches. No records were identified from other sources, such as reference lists.

In accordance with the literature and the review’s objective, the searches were systematically performed and organised into the PEO (Population, Exposure, Outcome) framework (see Table 1). Examples of search terms employed included “terminal care” OR “end of life care” for palliative care, “home health care” OR “home care” for home care, and “patient view,” “patient experiences” OR “family perspective” for perspectives and experiences. Moreover, the searches were adapted to each database, for instance, by incorporating Mesh terms or subject headings. The complete search history, including all terms and combinations used, is presented in Table S1.

**Table 1.** Search Terms Based on the PEO Framework and Related Inclusion and Exclusion Criteria.

PEO	Search terms	Inclusion criteria	Exclusion criteria
Population	Patients and family carers	Adult patients or adult patients’ and family carers’	Children, teenagers <18 years of age
Exposure	Palliative care AND care in the patient’s own home	Palliative care at home	Palliative care in a hospice, hospital or other inpatient settings
Outcome	Experiences	Experiences of palliative care at home	Other related persons’ (family or friends) or professionals’ experiences
Type of studies	Not Applicable	Qualitative studies or mixed methods (the qualitative part) in English, or the Nordic languages Swedish, Danish or Norwegian published between 2014 and 2025	Quantitative studies Review documents Commentaries Case studies

Inclusion criteria were (a) empirical qualitative studies exploring (b) adult patients or adult patients’ and family carers’ perspectives, (c) reported healthcare service as non-specialised or specialised palliative home care, and (d) published 2014 to 2025. Time frame was chosen to reflect contemporary palliative care practices and experiences. Furthermore, findings before 2014 have already been comprehensively synthesised in a previous review (Sarmento et al., 2017). Exclusion criteria were (a) patients and family carers younger than 18 years old, (b) studies exclusively reporting family carers’ perspective, (c) studies reporting experiences of unmet palliative care needs due to lack of palliative care, and (d) reported health care in in-patient settings, such as hospitals and hospices.

**Figure 1.**
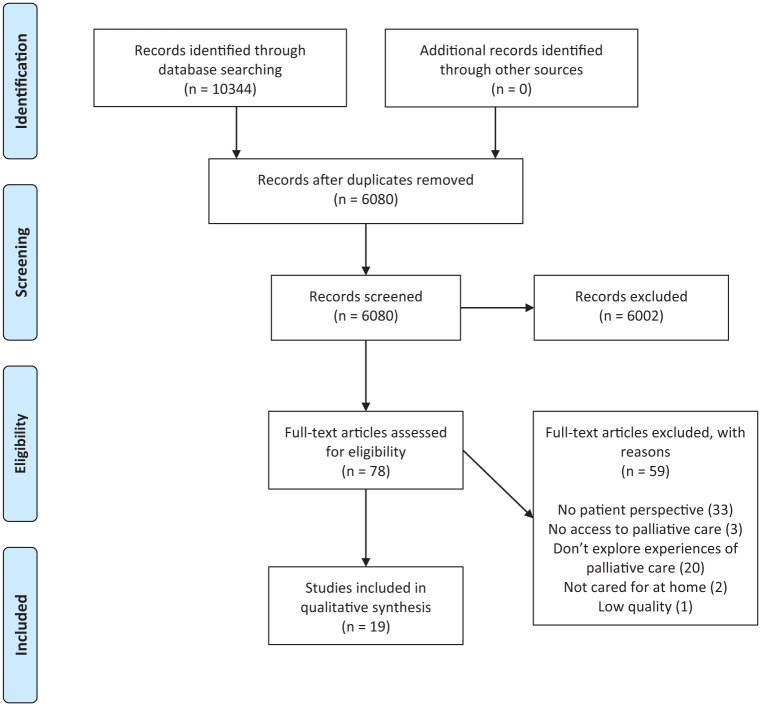
PRISMA 2009 flow diagram.

After removing duplicates, the searches yielded a total of 6,080 records (see, Figure 1). Duplicate records were identified and removed using the DedupEndNote online tool. All titles and abstracts were independently screened in Rayyan (Ouzzani et al., 2016) by at least two of the five authors and one additional colleague (LP, LC, MB, SN, JÖ, JK), who proceeded to evaluate the abstracts and select relevant articles. Any disagreements regarding the inclusion of articles were resolved by convening all authors during both the screening and evaluation.

### Appraisal

The quality assessment of the eligible full-text citations was conducted in accordance with the established checklist for qualitative studies, as outlined by the National Institute for Health and Care Excellence [NICE] (NICE, 2012). Accordingly, all 19 included articles were rated as good quality, ++ (where all or most of the checklist criteria were met, the conclusions are very unlikely to change) or + (where some of the checklist criteria were met, the conclusions are unlikely to change if they were not met or not adequately described). One study was excluded due to low quality, as the results were insufficiently described and lacked the analytical depth required to support a credible interpretation. The quality appraisal is presented in Table 2.

**Table 2.** Overview of Included Studies.

Author (year), country	Aim	Data-collection	Analysis	Participants	Setting	Main findings	NICE checklist
Aoun et al. (2016) Australia	Describe the lived experiences of older people coping with terminal cancer and living alone, focusing on how they face challenges of the biographical life changes from their disease progression	Individual semi-structured interviews	Thematic analysis	43 patients with cancer (age range 52–91 years, mean 74 years; 21 females, 22 males)	Home hospice care	Older people living alone at home with terminal cancer face challenges by adjusting to change, preserving normality, redefining normality and facing the end.	++
Dillen et al. (2021) Germany	To determine the key components contributing to a sense of security and how they relate to each other as experienced by patients and family caregivers in specialist and generalist palliative home care	Individual semi-structured interviews	Qualitative content analysis	215* patients with cancer, pulmonary, cardiovascular, neurological or other life-threatening diseases. (age range 29–98 years, mean 70 ± 13 years; 124 females, 91 males) 10 family carers (age range 50–77; 7 females, 3 males)	Generalist and specialist palliative home care services	Patients and carers emphasised the importance of a sense of security in palliative home care, which was most strongly associated with availability, patient-centredness, professional competence, and trust. Specialist palliative home care was perceived as particularly effective due to comprehensive responsibility, collaboration, and direct communication.	+
Dobrina et al. (2016) Italy	To explore the needs and wishes of patients and their caregivers during the last week of life at home	Individual semi-structured interviews	Descriptive phenomenology	11 patients with advanced cancer (age range 48–75 years, mean 54 years: 5 females, 4 males) 11 family carers (age range 20–75 years, mean 50 years; 8 females, 3 males)	Home-palliative cancer care service	Patients and family caregivers experienced the final phase of life as a complex process, marked by both a longing for death and a desire for independence. Unshared worries and fears negatively affected quality of life, while the close bond between patients and caregivers highlighted the need to view them as a unit of care.	++
Driller et al. (2024) Norway	To acquire insights into the experiences of advanced seriously ill cancer patients at home while undergoing palliative treatment and engaging in ACP in primary healthcare settings	Individual, semi- structured interviews	Reflexive thematic analysis	12 patients with cancer (age range 55–81 years, mean 72 years; 6 females, 6 males)	Primary care/community home-care services	The ability to preserve normality and receive compassionate, understanding care helped patients and families feel secure and in control at home. Advance care planning and open discussions about the future further strengthened trust and preparedness for end-of-life care.	++
Hardy et al. (2014) UK	To explore how patients and spouse-carers manage their involvement with care professionals in the community setting	Individual semi- structured interviews	Phenomenological template analysis	8 patients with advanced disease (age range 60–76 years; 3 females, 5 males) 8 family carers, spouses (age range 62–68 years; 5 females, 3 males)	Community health-care services	Patients and spouse-carers were interdependent in coordinating care and managing relationships with healthcare professionals. Together they actively adapted their strategies to manage care at home based on shared experiences.	++
Hermosilla-Ávila et al. (2021) Chile	To explore the meaning of palliative care as perceived by the family caregiver and the patient with advanced cancer	In-depth interviews with patient-family dyads and participant observation during home nursing visits	Hermeneutic phenomenological approach	7 patients with cancer (6 aged 30–64, 11 aged 65+; 10 females, 7 males) 17 family carers (4 aged 30–64, 13 aged 65+; 15 females, 2 males)	Hospital based palliative home care	Patients and family carers emphasised the importance of developing empathic relationships with nursing staff to meet emotional needs and promote well-being. The study highlighted the need for collaborative and interprofessional work to provide holistic and compassionate palliative care.	+
Jack et al. (2016) UK	To explore patients’ and family caregivers’ experiences and perceptions of Hospice at Home care	Individual or joint, semi-structured interviews	Thematic analysis	16 patients with cancer or other life-threatening disease (aged 61–90 years; 6 females, 10 male) 25 family carers (age range 41–90 years; 19 females, 6 males)	Hospice at home service	Holistic and person-centred care, including open communication, caregiver support, and respect for choice, promoted patients’ and families’ emotional, social, and physical well-being at home.	+
Klarare et al. (2017) Sweden	To investigate how the work of the team is manifested in care episodes narrated by patients and families receiving specialised palliative home care.	Narrative research interviews	Thematic analysis	6 patients and 7 family carers (age range 50–89 years, 8 females, 5 males)**	Specialised palliative home care	Experiences of security and continuity were enhanced through the palliative care team’s 24/7 availability, sensitivity, and flexibility in meeting patients’ and families’ needs. A holistic team approach, with personal knowledge of each family’s situation, enabled trust and continuity of care at home.	++
Klarare et al. (2018) Sweden	To describe patients’ and family caregivers’ experiences of specialist palliative home care team actions that are identified by the participants as helping or hindering interventions.	Individual interviews, critical incident technique	Critical incident technique	6 patients and 7 family carers (age range 50–89 years, 8 females, 5 males)**	Specialised palliative home care	Patients and family carers experienced reliability, keeping promises, and partnership with specialist palliative care teams as essential in meeting their needs and maintaining daily life at home. Mutual trust, respect, and inclusion in care decisions were described as features that enable supportive and dignified home care.	++
Mousing et al. (2018) Denmark	To explore the experiences of patients with COPD living in their own home in relation to communication with professional caregivers in home care about everyday life with COPD	Individual semi-structured interviews.	Qualitative descriptive analysis	12 patients with moderate to severe COPD (age range 53–90, mean 70, 3 years; 8 females, 4 males)	Community home care services	Continuity in professional caregivers and proactive conversations about illness progression, future life, and dying enabled trust and emotional support for patients with COPD receiving care at home.	++
Nysæter et al. (2022) Norway	To explore the preferences for home care over time to enable home death among adult patients with cancer in the late palliative phase	Individual interviews	Grounded theory	9 patients (age range 47–90 years, mean 71 years; 2 females, 7 males)	Support from cancer care coordinators, and they could receive community home care.	Patients’ preference to die at home was grounded in hope and trust in receiving the care they needed. Reliable, compassionate, and competent healthcare personnel, together with timely, continuous, and adaptive organisation of care, enabled safety, autonomy, and the ability to live meaningfully until death.	+
Oosterveld-Vlug et al. (2019) The Netherlands	To gain insight into the elements that patients with advanced cancer and relatives consider essential for high-quality palliative care, and whether these essentials are present in the actual care they receive.	Individual semi structured interviews	Thematic analysis	13 patients with advanced cancer (aged 58–86 years; 4 females, 9 males) 14 family carers (aged 40–79 years; 11 females, 3 males)	Generalist palliative care (GP and community nursing staff)	High-quality palliative home care was enabled by accessible, person-centred, and proactive professionals, supported by clear procedures and effective communication across services. Gaps in collaboration and information transfer risked compromising care, particularly for patients without assertive family support.	++
Ora et al. (2023) Australia	To evaluate a nurse-led model of supportive care in a COPD outpatient service from patient and caregiver perspectives.	Individual semi structured interviews	Qualitative content analysis	12 patients who had received nurse-led COPD supportive care (age range 53–80 years, mean 69 years; 5 females, 7 males) 7 family carers (age range 45–82 years, mean 67; 6 females, 1 male)	Nurse-led COPD supportive care in the outpatient service	Helpful aspects of COPD supportive home care included guidance in symptom management, participation in advance care planning, home visits, expert advice, continuity and trust, caring relationships, and caregiver support. These elements contributed to a more effective and person-centred model of care.	++
Staats et al. (2025) Norway	To explore the complex process of death acceptance as experienced by patients receiving palliative care at home.	Individual semi structured interviews	Hermeneutic analysis Secondary analysis	13 patients with diagnosis of a life limiting illness in the palliative phase (age range 55–94 years, mean 75 years; 8 females, 5 males)	General practitioner, community home care services, and support from cancer coordinator. 6 patients had support from a specialised palliative care team.	Supportive and trusting relationships with family and healthcare professionals were crucial for patients’ acceptance of death and maintenance of quality of life at home. Familiar surroundings, honesty, and open conversations about death promoted peace, meaning, and reconciliation during the end-of-life process.	+
Söderberg et al. (2015) Sweden	To describe the experience of security and insecurity in patients with chronic heart failure who were treated by Advanced Home Care in a city in Sweden	Individual semi structured interviews	Qualitative content analysis	8 patients with heart failure (age range 59–91 years, mean 86 years, 5 females, 3 males)	Advanced palliative home care	A multidisciplinary team approach, attentive nursing, and a strong focus on the patient’s perspective were essential features for creating a sense of security in palliative home care.	+
Sønning and Fossum (2020) Norway	To investigate how at-home cancer patients perceived their life situations during the palliative care phase.	In-depth individual interviews	Phenomenological analysis	8 patients with different types of cancer (age range 35–63 years: 6 females, 2 males)	Not specified (Palliative care at home)	Living with uncertainty, being seen and heard by healthcare professionals, and managing daily challenges shaped patients’ experiences of meaning, suffering, and relief in palliative home care.	+
Svensson and Wåhlin (2018) Sweden	To describe patients’ perceptions of what it is like to be cared for by a specialised palliative care team within hospital-based palliative home care.	Individual semi structured interviews	Phenomeno-graphical approach	14 patients, 13 with cancer, 1 with other life-threatening diagnosis (age range 42–95 years, mean 73 years; 10 females, 4 males)	Hospital-based palliative home care	Patients felt a sense of safety through the specialised palliative care team’s 24/7 telephone support. Receiving care at home reduced hospitalisations and was perceived as safe and dignified, underscoring the effectiveness and value of providing palliative care in the patient’s own environment.	+
Talabani et al. (2020) Sweden	To describe patients’ experiences of a new model of person-centred integrated heart failure and palliative care at home.	Individual semi structured interviews	Qualitative content analysis	12 patients with severe heart failure (age range 63–96 years, mean 81 years; 4 females, 8 males)	Integrated palliative advanced home care and heart failure care clinical care (part of an intervention study, the PREFER intervention)	Patients felt secure and safe through readily available, continuous, and trustworthy care at home. Being acknowledged as both a person and a patient through person-centred, integrated heart failure and palliative care created a holistic and supportive care environment.	++
Wennman et al. (2020) Sweden	To explore the experiences of receiving advanced home care among patients affected by life-threatening illness and their close relatives.	Unstructured in-depth interviews, individual or in dyads	Qualitative content analysis	11 patients affected by life-threatening illness (aged 53–87, 3 females, 8 males) 8 family carers (age and gender not reported)	Advanced palliative home care (cooperation between municipal and in-patient care, provided 24 hr per day, 7 days per week)	Patients and relatives perceived advanced home care as safe and of high quality, highlighting the importance of early access, a secure environment, and person-centred care in managing care at home.	++

* Eighteen patients were unable to complete the interview, but age and gender are not reported for the group who participated. **Based on the same fieldwork. Age and gender not reported separately for patients and family carers in the original papers.

### Synthesis of Findings

The synthesis was informed by the principles of interpretive meta synthesis, particularly its focus on iterative interpretation and the abstraction generated from existing qualitative findings (Thorne et al., 2004). To begin with, we read and re-read the results of the included studies several times in order to become familiar with the data. Overall study components were compiled to provide an overview of the studies: authors and date of publication, country, population and number of participants, aim of the study, data collection and analysis methods and finally, the main findings (Table 2).

Using the 6S model as an analytical lens (Österlind & Henoch, 2021), we adopted an abductive approach (Lipscomb, 2012) to synthesise the patients’ and family carers’ experiences of palliative care at home. NVivo was used to extract and organise data from the original articles included in the review. This was done through line-by-line coding, conducted by the first author (LP), with a focus on data segments relevant to the study’s aim. Each relevant segment was assigned a primary code based on the 6S framework. When data could not be clearly coded with any of the predefined 6S codes, new codes were created inductively. Thereafter, LP once again thoroughly read and re-read the text within each group of codes to further refine the analytical approach. The coded data were iteratively reviewed, compared, and grouped by all authors to reach consensus and develop seven descriptive themes (Thomas & Harden, 2008). Guided by the research question, the synthesis evolved from a descriptive level of interpretation to a higher level of conceptual abstraction. The initially synthesised themes were then thoroughly examined and discussed by all authors to ensure consistency and depth of interpretation. Through this iterative process, the themes were further refined, leading to the generation of three analytical themes representing enablers that facilitate palliative care at home, as reflected by patients’ and family carers’ perspectives (LP, MB, SN, JÖ, JK).

## Results

A total of 19 studies were included in the synthesis of the literature on the experiences of 422 patients and 107 family carers enrolled in specialised and non-specialised palliative home care services. Nine studies explored patient experiences, while 10 addressed the experiences of both patients and family carers. All studies used qualitative methods and generated data through individual interviews or dyadic interviews with patients and family carers. A range of methodological and analytical approaches were applied in the studies, including thematic analysis (5), content analysis (5), different phenomenological approaches (4), phenomenography (1), descriptive analysis (1), grounded theory (1), hermeneutic analysis (1), and critical incident technique (1). Two of the studies included used data from the same field work. Most studies were conducted in Europe, including Germany (1), Italy (1), The Netherlands (1), and the UK (2), and particularly in Scandinavia: Denmark (1), Norway (4), Sweden (6). Studies were also conducted in Australia (2) and Chile (1). The described palliative home care services were referred to as hospice care, advanced palliative home care, palliative nursing, and community-based care. See Table 2 for an overview of included studies.

The results are structured around three interrelated enablers that together demonstrate the features of palliative care facilitating care at home, as reflected by patients’ and family carers’ experiences. Together, these three enablers (*accessible palliative care*, *building trusting relationships* and *everyday life at home*) interact for patients and family carers to feel safe and supported at home. See Table 3 for an overview of the results.

**Table 3.** Enablers and Practices in Palliative Care That Facilitate Home Care, as Illustrated by Patients’ and Family Carers’ Experiences.

Enablers	Practices		
To feel safe and supported at home			
Enabling accessible palliative care	Being reliable and accessible – regular contact through visits and phone calls	Providing fundamental care, supporting everyday household tasks, and making practical home adjustments	Guiding family carers to provide or assist with care
Enabling trusting relationships	Being well informed and collaborate in the care team	Being present, listening, conversing, and adapting to daily changing needs	Encourage conversations about both daily life and existential issues
Enabling everyday life at home	Acknowledging the patient’s autonomy and control within their own home	Include both patient and family carer in the care team	Adopting a person-centred approach that recognises and responds to the needs of both patients and family carers

### Enabling Accessible Palliative Care

This type of enabler refers to experiences related to daily challenges faced by patients and family carers as related to symptom distress and the significance of symptom relief, emphasising the importance of enabling accessible palliative care at home. Severely distressing symptoms, such as pain or dyspnoea, caused anxiety for both patients and family carers (Dobrina et al., 2016; Mousing et al., 2018; Söderberg et al., 2015). Some patients felt overwhelmed by the burden of the illness and severe symptoms raised fear of a painful death (Dobrina et al., 2016; Hermosilla-Ávila et al., 2021; Mousing et al., 2018). As the disease progressed and physical deterioration became more apparent, patients began to question whether they could remain at home, which triggered feelings of sadness, frustration and diminished self-worth (Driller et al., 2024).

When palliative care was provided in the home, it was important for patients and their family carers to feel they could rely on receiving the help they needed (Klarare et al., 2018; Nysæter et al., 2022; Wennman et al., 2020). Patients and family carers valued the multidisciplinary expertise of the healthcare professionals, describing them as knowledgeable and responsive in monitoring health status, managing symptoms, and providing practical guidance in complex or uncertain situations (Dillen et al., 2021; Jack et al., 2016; Nysæter et al., 2022; Ora et al., 2024; Svensson & Wåhlin, 2018; Wennman et al., 2020). Trusting the healthcare professionals’ ability to offer competent support made them feel more secure (Svensson & Wåhlin, 2018; Talabani et al., 2020). Furthermore, the availability of healthcare professionals when palliative care was needed created a safe supportive environment for both patients and family carers, reassuring them that help was always at hand (Svensson & Wåhlin, 2018; Wennman et al., 2020). This availability was experienced through being able to contact palliative care professionals at any time, rely on a timely response, and receive home visits when needed, which patients and family carers described as essential for feeling safe and supported (Hermosilla-Ávila et al., 2021; Klarare et al., 2017; Söderberg et al., 2015; Svensson & Wåhlin, 2018; Talabani et al., 2020; Wennman et al., 2020). A sense of security was further strengthened through regular contact, such as visits and phone calls, combined with continuity of care (Söderberg et al., 2015; Talabani et al., 2020).

While access to care was important, achieving a sustained sense of security required a flexible and responsive healthcare system capable of adapting to patients’ changing needs, as limited adaptability often gave rise to feelings of insecurity and disappointment (Nysæter et al., 2022). When the care team was unavailable, patients often refrained from seeking help (Dillen et al., 2021; Hermosilla-Ávila et al., 2021). In the absence of accessible out-of-hours support, patients sometimes felt they had no choice but to call an ambulance, which highlighted a gap in the continuity of receiving accessible palliative care services (Ora et al., 2024).

Enabling accessible palliative care also played a crucial role in relieving the pressure on family carers, whose presence was often required around the clock (Dobrina et al., 2016). For some family carers, participating in the patient’s care was important. This was enabled by healthcare professionals who taught and guided family carers to provide or assist with care (Dillen et al., 2021; Jack et al., 2016). Furthermore, healthcare professionals’ initiative in assisting with fundamental care needs, household tasks, and practical adjustments in the home was highly appreciated, as it not only supported patients but also alleviated some of the burden experienced by family carers (Dillen et al., 2021; Jack et al., 2016; Klarare et al., 2017; Ora et al., 2024).

Living with a life-threatening disease meant having to navigate the healthcare system, which affected both patients and family carers (Hardy et al., 2014; Wennman et al., 2020). Family carers were often responsible for organising and coordinating services to ensure that the patient’s care needs were met, a task that could be complex and overwhelming (Hardy et al., 2014). Both patients and family carers reported that healthcare professionals, and nurses in particular, played a crucial role in supporting families through this process (Jack et al., 2016). However, any lack of communication between the different healthcare professionals often meant patients and family carers had to assume a more active role in coordinating care (Oosterveld-Vlug et al., 2019; Wennman et al., 2020).

### Enabling Trusting Relationships

This type of enabler relates to experiences in regard to social relationships, synthesis and strategies. It highlights how continuous and trusting relationships with healthcare professionals shaped patients’ and family carers’ experiences of palliative home care, emphasising the importance of social connection, communication, and professional reliability in promoting safety and support. Since receiving care at home meant having healthcare professionals visit around the clock, it was important to build trusting relationships, which contributed to the psychological well-being of patients and family carers (Dillen et al., 2021; Jack et al., 2016; Söderberg et al., 2015; Wennman et al., 2020).

Trustful relations were built through continuity, clear communication, and reliability, compassion, and competence of healthcare professionals (Dillen et al., 2021; Nysæter et al., 2022; Söderberg et al., 2015; Talabani et al., 2020). Patients often valued support from healthcare professionals who were familiar with them and their home routines, as it made it easier for staff to interpret non-verbal cues and emotional expressions, which in turn enhanced patients’ sense of security (Mousing et al., 2018; Svensson & Wåhlin, 2018). Continuity in care was also related to how members of the palliative care team collaborated, meaning they had to be well-informed about and familiar with the patients (Klarare et al., 2017). Having palliative care provided by a multi-professional team who cooperated closely was greatly valued by patients and their families (Dillen et al., 2021; Svensson & Wåhlin, 2018; Talabani et al., 2020). Moreover, when such a sense of security and trust had been established, patients and family carers sometimes perceived the healthcare professionals as extended family (Svensson & Wåhlin, 2018). In contrast, when conditions for building relationships with healthcare professionals were lacking, for example, poor communication and visits from unfamiliar staff, this could trigger frustration and stress (Hermosilla-Ávila et al., 2021; Mousing et al., 2018; Svensson & Wåhlin, 2018).

For both patients and family carers, the illness served as a reminder of life’s finitude and the inevitability of separation through death. While strong and supportive connections provided comfort and reconciliation, they also heightened the sorrow of impending separation (Nysæter et al., 2022; Söderberg et al., 2015; Staats et al., 2025). Family and friends were important for patients, as they provided both emotional support and practical assistance (Aoun et al., 2016; Driller et al., 2024; Hardy et al., 2014; Söderberg et al., 2015; Staats et al., 2025). Both patients and family carers found it easier to cope with challenges when they had someone close to share them with, while family carers experienced greater strain when left alone in care responsibilities (Aoun et al., 2016; Dobrina et al., 2016). Healthcare professionals supported patients and family carers through their presence and relationships, offering valued social and intellectual stimulation, which were especially valued as health declined and social interactions became limited (Aoun et al., 2016; Jack et al., 2016; Svensson & Wåhlin, 2018; Talabani et al., 2020). Patients appreciated the opportunity to talk about more than their illness, about everyday life, and valued positive relationships with healthcare professionals (Hardy et al., 2014; Svensson & Wåhlin, 2018).

Patients and family carers found it burdensome not knowing what to expect and not feeling prepared for the death. Some patients expressed fear and uncertainty about the future and death, expressing concern for their loved ones after their passing (Dobrina et al., 2016; Mousing et al., 2018). Patients coped with the uncertain future in different ways, for example, by suppressing thoughts, focusing on everyday life, seeking meaning in life after death or preparing for it. Others expressed a need to process their thoughts and emotions verbally (Aoun et al., 2016; Mousing et al., 2018; Söderberg et al., 2015; Staats et al., 2025). Patients established meaningful relationships with healthcare professionals, which gave them the confidence to openly discuss their illness and thoughts about the future (Jack et al., 2016; Nysæter et al., 2022; Staats et al., 2025). Healthcare professionals initiating conversations about existential concerns was seen as desirable, as such dialogues supported relief and well-being (Jack et al., 2016; Mousing et al., 2018; Svensson & Wåhlin, 2018). Trust and open conversations about death and future care were supported when healthcare professionals respected patients’ autonomy, recognised them as individuals, and invited them to participate in decisions about their care (Nysæter et al., 2022). However, some patients did not want to be a burden to the healthcare professionals and therefore did not talk about what they perceived as sensitive issues (Mousing et al., 2018).

### Enabling Everyday Life at Home

This type of enabler relates to meanings of home as the preferred place of care, as related to maintaining self-image and self-determination. It also relates to how palliative care supported patients and family carers in maintaining a meaningful daily life, marked by a sense of independence. For patients, remaining at home and maintaining an everyday life was reported as desirable, as it created a sense of security and allowed them to feel in control of their own lives (Aoun et al., 2016; Dobrina et al., 2016; Driller et al., 2024; Nysæter et al., 2022; Söderberg et al., 2015; Svensson & Wåhlin, 2018). For patients it was important to have control of their situation, as feeling of being out of control was associated with a sense of insecurity (Driller et al., 2024; Söderberg et al., 2015).

Some patients equated living with a life-threatening illness with having to give up their daily life and sense of identity (Aoun et al., 2016; Sønning & Fossum, 2020; Wennman et al., 2020). Patients found it burdensome when the illness reduced their independence, and some felt like a burden to their families, particularly when they were unable to fulfil their parental roles or contribute to household responsibilities (Aoun et al., 2016; Dobrina et al., 2016; Driller et al., 2024; Sønning & Fossum, 2020). Even when patients preferred to be at home, some were concerned that if their condition worsened it might be too much of a burden for their family carer (Driller et al., 2024). As the illness progressed, patients often had to re-evaluate what mattered to them most, as they could no longer engage in activities that had previously defined them (Dobrina et al., 2016). Maintaining even small elements of independence, such as getting up and caring for themselves in the morning, was described as vital for preserving a sense of normality and control (Nysæter et al., 2022). A perception of maintaining independence and a sense of self was maintained by redefining normality and accepting formal support (Aoun et al., 2016; Driller et al., 2024).

The home environment was described as safe, familiar, and supportive, enabling patients to maintain daily routines, stay close to loved ones, and care for themselves as much as possible, contributing to a sense of normalcy and helping to preserve their autonomy (Driller et al., 2024; Nysæter et al., 2022; Söderberg et al., 2015; Staats et al., 2025; Svensson & Wåhlin, 2018). Engaging in everyday activities within this setting brought satisfaction and was said to contribute to both emotional well-being and overall quality of life (Dobrina et al., 2016; Driller et al., 2024; Staats et al., 2025).

Being cared for as a person and not merely as patients or carers, as well as feeling genuinely acknowledged by healthcare professionals, was highly valued (Klarare et al., 2018; Nysæter et al., 2022; Oosterveld-Vlug et al., 2019; Ora et al., 2024; Svensson & Wåhlin, 2018; Talabani et al., 2020; Wennman et al., 2020). A person-centred approach was perceived as a unique feature of palliative care, with healthcare professionals demonstrating an understanding of patients’ and family carers’ needs (Jack et al., 2016; Klarare et al., 2017; Klarare et al., 2018; Svensson & Wåhlin, 2018).

Having care provided in their own home was associated with open communication, which reduced strain and helped patients feel more like a person (Ora et al., 2024; K. Staats et al., 2025; Svensson & Wåhlin, 2018). Patients and family carers emphasised that meaningful conversations with healthcare professionals and being listened to and included as part of the care team were essential to keeping patients’ wishes and needs at the centre of care (Jack et al., 2016; Klarare et al., 2018; Sønning & Fossum, 2020; Talabani et al., 2020). It was also important that the healthcare professionals respected the home as a place where the patient was in charge (Nysæter et al., 2022; Svensson & Wåhlin, 2018). When patients and family carers felt included in the care team and received clear explanations from healthcare professionals, their sense of involvement was strengthened, thus enabling them to maintain everyday life (Klarare et al., 2017; Klarare et al., 2018; Talabani et al., 2020). Supportive communication and knowledge-sharing from palliative care professionals enabled patients and family carers to better understand their situation and manage many aspects of care on their own (Dillen et al., 2021; Ora et al., 2024). Conversely, a lack of such communication led to feelings of fear and insecurity (Dillen et al., 2021).

## Discussion

Our meta-synthesis demonstrates that palliative home care is shaped through a dynamic interaction between enablers and practices, encompassing accessible palliative care, trusting relationships, and enabling everyday life at home. Together, these findings deepen the understanding of how palliative home care enables patients and family carers to feel safe and supported at home despite a progressing illness. Our meta-synthesis is consistent with previous research on palliative home care, which has emphasised key elements, such as availability, home visits, effective symptom management and strong communication skills in contributing to a sense of security at home (Sarmento et al., 2017). In addition, our meta-synthesis extends previous knowledge by demonstrating that these aspects are relevant in both specialist and generalist palliative home care and contributes with factors and practices that enable home care from the perspective of patients and family carers. It further contributes with a deepened understanding of the significance of safety and security for patients and family carers in home care, which is not emphasised in the 6S model’s conceptualisation. Both the previous review (Sarmento et al., 2017) and our meta -synthesis indicate that both the home as place of care and access to palliative care play a decisive role in shaping patients’ and family carers’ experiences.

Our meta-synthesis shows that enabling accessible palliative care round-the-clock was identified as a key factor in promoting a sense of safety and reassurance among patients and family carers. Being able to get support from healthcare professionals 24/7 was also highlighted by Sarmento et al. (2017), who describe how this enabled patients and family carers to feel secure and supported at home. Access to palliative services in the home setting has also been suggested to play a central role in supporting patient outcomes, providing care aligned with patients’ preferences and reducing hospital utilisation (Pask et al., 2025; Pinto et al., 2024). Our meta-synthesis demonstrates that, when palliative care was accessible, patients and family carers perceived the care teams as possessing the necessary expertise to proactively anticipate, identify and manage disease-related issues in a timely and competent manner. In contrast, a lack of access to palliative care services when needed can have serious and wide-ranging consequences for patients and family carers alike, affecting their well-being, sense of security and ability to cope (Aebischer Perone et al., 2018; Fjose et al., 2018). Highly accessible palliative care services thus emerge as a fundamental prerequisite for ensuring high-quality palliative care at home.

Access to palliative care remains unequally distributed, both in Sweden (Larsdotter et al., 2024) and globally, with disparities linked to factors such as age, gender, diagnosis, socioeconomic status, and geographic location (Abel & Kellehear, 2016; Peeler et al., 2025; WHO, 2021). Even within countries known for having nationally implemented palliative care, structurally vulnerable populations often face barriers that limit their access to palliative care services (Peeler et al., 2025; K. I. Stajduhar et al., 2019). Moreover, healthcare services are not always tailored to the specific needs of patients and family carers, particularly of older individuals, for whom fragmented and poorly coordinated care may compromise continuity, increase caregiver burden, and undermine their sense of security and sense of being at home (Barker et al., 2023; Fjose et al., 2018; Saarnio et al., 2016).

Social relationships have been described as encompassing the value of connections with family carers and of other close ones, along with the crucial importance of building trusting and meaningful relationships with healthcare professionals (Österlind & Henoch, 2021). Our meta-synthesis demonstrated that enabling trusting relationships with healthcare professionals was important for patients and family carers to feel safe and supported in the context of palliative home care. Likewise, Bertaud et al. (2025) highlight the importance of relationships in palliative care, where care is grounded in relational ways of being with patients and families, characterised by responsiveness, empathy, respect and hope. Our meta-synthesis illustrates how healthcare professionals establish trusting relationships by providing continuity of care, clear communication, reliability, compassion and competence. These findings emphasise the importance of enabling trusting relationships in palliative home care. This requires organisational structures that facilitate continuity, allow time for relational engagement and encourage interprofessional collaboration.

Our meta-synthesis shows that establishing trustful relationships is essential for open discussions about existential concerns, with patients preferring healthcare professionals to initiate these conversations. Healthcare professionals should therefore take an active role in building trust and initiating these discussions to support patients in expressing their thoughts and emotions at the end of life.

Our meta-synthesis indicated that enabling everyday life at home and making it a safe and secure place was important, as many patients appreciated remaining in their own homes. This aligns with previous research showing that both patients and family carers often identify the home as their preferred place of care and death (Fereidouni et al., 2021; Gomes et al., 2015; O’Sullivan et al., 2024). Feeling at home has been described as central to palliative care, as it enhances the sense of comfort and security for individuals receiving care in their own environment (Devik et al., 2015; Öhlén et al., 2002). Furthermore, home as a place of care has been described as a source of continuity and identity, promoting a sense of being oneself (Dekkers, 2009). Particularly among older people, home is associated with feelings of selfhood, connectedness to loved ones and familiar surroundings, and arises when past experiences and expectations are integrated with the present environment and relationships (Saarnio et al., 2016; Saarnio et al., 2018). Our meta-synthesis highlights that care in one’s own home was experienced by patients as promoting a stronger sense of personal identity and they perceived their homes as calm, secure and familiar spaces. This is consistent with Collier et al. (2015), who describe how home is a dynamic concept for people approaching the end of life, encompassing expressions of social and cultural identity, including symbolic and emotional connections, rather than just a physical place or address. Yet, this understanding does not apply to everyone. For people without stable housing, the notion of home may be fragile or absent (Håkanson & Öhlén, 2016; K. I. Stajduhar et al., 2019). Recognising these variations is crucial, as overlooking them can lead to palliative care privileging some while marginalising others (K. I. Stajduhar, 2020).

## Strengths and Limitations

Credibility is a central principle in qualitative interpretive meta-synthesis, requiring interpretations to be well-grounded, reflexive, and transparent (Thorne, 2022). In this review, credibility was enhanced through a transparent and systematic process, exemplified by the clear articulation of inclusion and exclusion criteria as part of an overall commitment to methodological transparency. The inclusion of both generalist and specialist palliative care services can be viewed as a strength in this synthesis, as it enabled a more nuanced understanding of how the needs of patients and family carers are addressed across different care contexts. This is important, as palliative care needs arise across various settings, and meeting them requires collaboration and integration between generalist and specialist services rather than restricting care to specialist contexts (Pereira et al., 2024). A limitation with this synthesis is that the type of healthcare systems framing the provision of palliative home care was not always clearly described in the included studies, which complicates the interpretation of how structural and contextual factors may have influenced patients’ and family carers’ experiences. This highlights the need for greater transparency regarding study contexts in future palliative care research.

A limitation is that all records were identified through electronic searches only; therefore, potentially relevant grey literature and studies from other sources may have contributed to the interpretations. To ensure that the primary studies included in the synthesis met appropriate standards of quality, the established NICE checklist for qualitative research (NICE, 2012) was applied. Rather than serving as a basis for exclusion, the quality appraisal informed a reflexive understanding of how methodological and ethical limitations might have shaped the original findings (Thorne, 2022). For instance, in some studies, the researchers’ roles were not clearly described, raising potential ethical concerns in cases where participants may have been in a dependent relationship with them. Moreover, in several studies, patient recruitment by caregivers may have introduced a positive bias in the reported experiences.

We adopted an abductive approach (Lipscomb, 2012) by using the 6S model as an analytical lens (Österlind & Henoch, 2021) which focused the synthesis on dimensions previously described as essential for person-centred palliative care from the perspectives of patients and family carers. One limitation of this approach could be that using the 6S model as an analytical framework may have led to results being limited to confirming the dimensions already defined in the model. The fact that the results highlight the importance of accessibility and place of care indicates that we have managed to achieve a balance in this process, as these aspects are not explicitly emphasised in the 6S model. The synthesis resulted in enablers and practices grounded in the perspectives of patients and family carers, which can be considered a strength, as it emphasises the person-centred approach of palliative care.

Eleven studies were performed in the Nordic countries, with the others in Australia, Chile, the Netherlands, Italy, Germany and Great Britain. Despite being conducted across different countries and continents, the studies reported similar experiences of patients and family carers receiving palliative home care, suggesting that many needs are shared across contexts. However, most of the included studies were conducted in high-income countries and many studies from low- and middle-income countries were excluded as they described unmet palliative care needs and lack of access to palliative care (e.g. Kaba et al., 2021; Sudhakar et al., 2021). Since most research on palliative care is concentrated in a few countries with similar cultural and socioeconomic contexts, not all perspectives are represented, which may limit the diversity and global relevance of the findings.

## Conclusion

In conclusion, our meta-synthesis contributes with knowledge about practices enabling patients and family carers to feel safe and supported in palliative home care. Access to palliative care is realised through reliable and accessible healthcare professionals who maintain regular contact, provide essential and practical support in the home, and guide family carers in caregiving tasks. Trustful relationships are enabled when healthcare professionals collaborate effectively, remain present and attentive to patients’ and families’ needs, and invite dialogue about both everyday life and existential concerns. Enabling everyday life at home depends on recognising it as a setting where patients and family carers are in charge, supporting the maintenance of daily life, and involving both patients and family carers in a person-centred approach to palliative care. In order for palliative care to truly meet the needs of patients and family carers, it is crucial to consider these perspectives when organising services, so that individuals can receive support and care in the place they wish to be. Future research should focus on developing and evaluating care approaches that respond to the practices enabling patients and family carers to feel safe and supported in palliative home care, while also incorporating healthcare professionals’ and societal perspectives to assess feasibility and practical implementation.

## Supplemental Material

sj-docx-1-gqn-10.1177_23333936261435388 – Supplemental material for Person-Centred Palliative Home Care From a Patient and Family Carer Perspective – A Qualitative Interpretive Meta Synthesis Supplemental material, sj-docx-1-gqn-10.1177_23333936261435388 for Person-Centred Palliative Home Care From a Patient and Family Carer Perspective – A Qualitative Interpretive Meta Synthesis by Lotta Pham, Malin Bengtsson, Stina Nyblom, Joakim Öhlén and Jeanette Källstrand in Global Qualitative Nursing Research
