# Supplementary material for: Person-Centred Palliative Home Care From a Patient and Family Carer Perspective – A Qualitative Interpretive Meta Synthesis
Source: Glob Qual Nurs Res. 2026 Apr 6;13:23333936261435388. doi: 10.1177/23333936261435388 (PMC13058193; doi:10.1177/23333936261435388)
Supplement: sj-docx-1-gqn-10.1177_23333936261435388 – Supplemental material for Person-Centred Palliative Home Care From a Patient and Family Carer Perspective – A Qualitative Interpretive Meta Synthesis [file sj-docx-1-gqn-10.1177_23333936261435388.docx]

Supplementary Table 1. Overview of searches in the databases

| Data base |  |
| --- | --- |
| **Pubmed**  Date of Search: 250407  Number of hits: 1,859 | (("Palliative Care"[MeSH Terms] OR "Palliative Medicine"[MeSH Terms] OR "Hospice and Palliative Care Nursing"[MeSH Terms] OR "Terminal Care"[MeSH Terms] OR "end of life"[Title/Abstract] OR "palliative"[Title/Abstract] OR "Terminal Care"[Title/Abstract] OR "hospice"[Title/Abstract]) AND 2014/01/01:2025/12/31[Date - Publication] AND (("Home Care Services"[MeSH Terms] OR "Home Health Nursing"[MeSH Terms] OR "Home Care Agencies"[MeSH Terms] OR "Home Nursing"[MeSH Terms] OR "nurses, community health"[MeSH Terms] OR "at home"[Title/Abstract] OR "home based"[Title/Abstract] OR "home care"[Title/Abstract] OR "Home Nursing"[Title/Abstract] OR "private home"[Title/Abstract]) AND 2014/01/01:2025/12/31[Date - Publication]) AND (("Family"[MeSH Terms] OR "Patients"[MeSH Terms] OR "Caregivers"[MeSH Terms] OR "patient*"[Title/Abstract] OR "famil*"[Title/Abstract] OR "carer*"[Title/Abstract] OR "next of kin"[Title/Abstract] OR "Kinship"[Title/Abstract] OR "caregiver*"[Title/Abstract] OR "spouses"[MeSH Terms] OR "spouse*"[Title/Abstract] OR "husband*"[Title/Abstract] OR "wife"[Title/Abstract] OR "married"[Title/Abstract]) AND 2014/01/01:2026/12/31[Date - Publication]) AND (("attitude"[MeSH Terms] OR "view*"[Title/Abstract] OR "experienc*"[Title/Abstract] OR "perspectiv*"[Title/Abstract] OR "attitude*"[Title/Abstract]) AND 2014/01/01:2026/12/31[Date - Publication])) AND (2014:2025[pdat]) |
| **Cinahl**  Date of Search: 250407  Number of hits: 1,569 | ( MH "Palliative Care" OR MH "Terminal Care" MH "hospice care" OR MH "hospice nursing" ) OR XB ( "end of life" OR "palliative" OR "terminal care" OR "hospice care" ) ) AND ( ( MH "Home Health Care" OR MH "Home Health Nursing" OR MH "Home Nursing" OR MH "Community health nursing" OR MH "Home health agencies" ) OR XB ( "at home" OR "home based" OR "home care" OR "home nursing" OR "private home" ) ) AND ( ( MH "Patients" OR MH "Hospice Patients" OR MH "caregivers" OR MH "family" ) OR XB ( "patient*" OR "family*" OR "Carers" OR "next of kin" OR "Kinship" OR “caregiver*” OR “spouses” OR “spouse*” OR “husband*” OR “wife” OR “married” ) ) AND ( MH "attitude" OR XB ( "view*" OR "experienc*" OR "perspectiv*" OR "attitude*" ) Limiters - Publication Date: 20140101-20251231 |
| **Scopus**  Date of Search: 250407  Number of hits: 2,001 | ( TITLE-ABS-KEY ( "Palliative Care" OR "Palliative Medicine" OR "Hospice and Palliative Care Nursing" OR "Terminal Care" OR "end of life" OR "palliative" OR "Terminal Care" OR "hospice" ) AND TITLE-ABS-KEY ( "Home Care Services" OR "Home Health Nursing" OR "Home Care Agencies" OR "Home Nursing" OR "nurses, community health" OR "Home Care Services" OR "Home Health Nursing" OR "Home Care Agencies" OR "Home Nursing" OR "nurses, community health" OR "Home Care Services" OR "at home" OR "home based" OR "home care" OR "Home Nursing" OR "private home" ) AND TITLE-ABS-KEY ( "Family" OR "Patients" OR "Caregivers" OR "patient*" OR "famil*" OR "Carers" OR "next of kin" OR "Kinship" OR caregiver* OR spouses OR spouse* OR husband* OR wife OR married ) AND TITLE-ABS-KEY ( "attitude" OR "view*" OR "experienc*" OR "perspectiv*" OR attitude* ) ) AND ( LIMIT-TO ( DOCTYPE , "ar" ) ) |
| **Embase**  Date of Search: 250407  Number of hits: 4,291 | 'palliative therapy'/exp OR 'palliative nursing'/exp OR 'terminal care'/exp OR 'end of life':ti,ab,kw OR 'palliative':ti,ab,kw OR 'terminal care':ti,ab,kw OR 'hospice':ti,ab,kw AND 'home care'/exp OR 'nurse'/exp OR 'at home':ti,ab,kw OR 'home based':ti,ab,kw OR 'home care':ti,ab,kw OR 'home nursing':ti,ab,kw OR 'private home':ti,ab,kw AND ('family'/exp OR 'patient'/exp OR 'caregiver'/exp OR 'patient*':ti,ab,kw OR 'famil*':ti,ab,kw OR 'carer*':ti,ab,kw OR 'next of kin':ti,ab,kw OR 'kinship':ti,ab,kw OR 'caregiver*':ti,ab,kw OR 'spouse'/exp OR 'spouse*':ti,ab,kw OR 'husband*':ti,ab,kw OR 'wife':ti,ab,kw OR 'married':ti,ab,kw) AND ('home care'/exp OR 'nurse'/exp OR 'at home':ti,ab,kw OR 'home based':ti,ab,kw OR 'home care':ti,ab,kw OR 'home nursing':ti,ab,kw OR 'private home':ti,ab,kw) AND ('attitude'/exp OR 'view*':ti,ab,kw OR 'experienc*':ti,ab,kw OR 'perspectiv*':ti,ab,kw OR 'attitude*':ti,ab,kw) AND ('palliative therapy'/exp OR 'palliative nursing'/exp OR 'terminal care'/exp OR 'end of life':ti,ab,kw OR 'palliative':ti,ab,kw OR 'terminal care':ti,ab,kw OR 'hospice':ti,ab,kw) AND 'article'/it AND (2014:py OR 2015:py OR 2016:py OR 2017:py OR 2018:py OR 2019:py OR 2020:py OR 2021:py OR 2022:py OR 2023:py OR 2024:py OR 2025:py) |
| **Psycinfo**  Date of Search: 250407  Number of hits: 624 | ( MH "Palliative Care" OR MH "Terminal Care" MH "hospice care" OR MH "hospice nursing" ) OR XB ( "end of life" OR "palliative" OR "terminal care" OR "hospice care" ) ) AND ( ( MH "Home Health Care" OR MH "Home Health Nursing" OR MH "Home Nursing" OR MH "Community health nursing" OR MH "Home health agencies" ) OR XB ( "at home" OR "home based" OR "home care" OR "home nursing" OR "private home" ) ) AND ( ( MH "Patients" OR MH "Hospice Patients" OR MH "caregivers" OR MH "family" ) OR XB ( "patient*" OR "family*" OR "Carers" OR "next of kin" OR "Kinship" OR “caregiver*” OR “spouses” OR “spouse*” OR “husband*” OR “wife” OR “married” ) ) AND ( MH "attitude" OR XB ( "view*" OR "experienc*" OR "perspectiv*" OR "attitude*" ) Limiters - Publication Date: 20140101-20251231 |
